# Analyze the toxicities of benzalkonium chloride as a COVID-19 disinfectant in physiological goldfish (*Carassius auratus*)

**DOI:** 10.14202/vetworld.2023.1400-1407

**Published:** 2023-07-04

**Authors:** Sisilia Rivanda Arianto, Fendi Aprian Syah, Luthfiana Aprilianita Sari, Ayu Lana Nafisyah, Sulastri Arsad, Nadirah Musa

**Affiliations:** 1 Program Study of Aquaculture, Faculty of Fisheries and Marine, Universitas Airlangga, Campus C Mulyorejo Street, Surabaya 60115 East Java, Indonesia; 2 Department of Aquaculture, Faculty of Fisheries and Marine, Universitas Airlangga, Campus C Mulyorejo Street, Surabaya 60115 East Java, Indonesia; 3 Institute of Marine and Environmental Sciences, University of Szczecin, ul. Mickiewicza 16a, 70-383 Szczecin, Poland; 4 Department of Fisheries Science and Aquaculture, Faculty of Fisheries and Food Science, Universiti Malaysia Terengganu 21030 Kuala Nerus, Terengganu, Malaysia

**Keywords:** color brightness, fisheries management, freshwater species, integrated multi-trophic aquaculture, mortality

## Abstract

**Background and Aims:**

Coronavirus disease-2019 (COVID-19) impacts increasing the use of disinfectants (benzalkonium chloride), which indirectly accumulate in water. The disinfectant accumulation in waters has been studied, but there has been no study of its impact on aquatic commodities, especially fish with a high sensitivity, one of which is goldfish (*Carassius auratus*). Benzalkonium chloride can potentially affect several body proteins, including the cytoskeleton, nervous and endocrine systems, and fish physiology. This study aimed to determine the impact of benzalkonium chloride as a disinfectant on the level of color brightness, growth, gill histopathology, and mortality in goldfish. This investigation provides input into the impact of using disinfectants to prevent COVID-19 on aquatic commodities.

**Materials and Methods:**

This study utilized goldfish specimens sourced from Tulungagung, East Java, Indonesia. The experiment involved different concentration levels of benzalkonium chloride: (T1) 0 mg/L, (T2) 0.03 mg/L, (T3) 0.06 mg/L, (T4) 0.09 mg/L, and (T5) 0.12 mg/L. The research data were subjected to an analysis of variance for analysis. In cases where significant differences were observed, Duncan’s test was conducted for color brightness, growth, and mortality data. Furthermore, if the gill histopathological data yielded significant differences, additional tests were applied (Kruskal–Wallis and Mann–Whitney test).

**Results:**

The findings of this study demonstrated significant differences (p < 0.05) in the level of color brightness, growth, gill histopathology, and mortality in goldfish in response to varying concentrations of benzalkonium chloride. The relationship between the length and weight of the goldfish was analyzed using regression coefficients (b values), which were determined as 4.86, −0.04, −0.2, 0.8, and −0.07, respectively. Notably, the brightness level in the T2 group exhibited positive color results with a hue value of 11.55°, while optimal growth was observed in the T4 group, as evidenced by b value of 0.8. The gill histopathological data showed significant differences (p < 0.05). The scoring of histopathological damage in the goldfish gills ranged from 0 to 10, with higher scores indicating more severe damage. The highest total score of 10 was observed in the T5 group exposed to a concentration of 0.12 mg/L, resulting in an 85% mortality rate. This indicates that benzalkonium chloride, with its toxic compounds, can disrupt the respiratory system of fish and lead to death.

**Conclusion:**

The effects of benzalkonium chloride were evident even at a concentration of 0.03 mg/L. With increasing concentration, there was an increase in mortality rate, a decrease in growth, and a rise in histopathological damage to the gills. These findings highlight the negative impact of using conventional disinfectants on water and its organisms, emphasizing the need for further research on environmentally friendly alternatives.

## Introduction

The 2019 pandemic was caused by the severe acute respiratory syndrome coronavirus 2 virus, also known as the Coronavirus disease-2019 (COVID-19). According to the World Health Organization, the use of disinfectants for cleaning is crucial in preventing the spread of viruses that can be found on surfaces [1]. Disinfectants are substances used to kill pathogens in the environment, but they can also pose a risk to non-target organisms, including living organisms, as highlighted by Prasetiya *et al*. [2]. It is important to strike a balance between effectively eliminating pathogens and minimizing potential harm to non-target organisms during the use of disinfectants. The global market witnessed an increase in the total number of disinfectant users from 0.66 billion in 2019 to 0.78 billion in 2020. Among the disinfectants recommended by the World Health Organization benzalkonium chloride is a chlorine-based disinfectant [3]. Benzalkonium chloride, which contains quaternary ammonium compounds, has the potential to negatively impact skin and respiratory health [4]. In Korea, benzalkonium chloride has been detected in freshwater at concentrations of up to 57 g/L, as reported by NIER [5]. Due to its resistance to degradation and the challenges associated with its detection in water, it is anticipated that the content of benzalkonium chloride in public waters in Indonesia may increase during the current pandemic [6].

Exposure to benzalkonium chloride significantly impacts various proteins involved in the cytoskeleton, nervous, and endocrine systems [7]. The stimulus from benzalkonium chloride exposure can affect the skin proteins through the nervous and endocrine systems, leading to changes in the color and brightness of fish scales [8]. Chemical and physiological stress induced by benzalkonium chloride can result in an increase in stress fibers containing actin, which hinders cell growth. Inadequate water conditions have a detrimental effect on fish growth as they increase susceptibility to acute or chronic stress [9]. The entry of liquid benzalkonium chloride into the gill organs can result in respiratory system disorders in fish due to the reduced supply of dissolved oxygen (DO) in the water. Histopathological examination of the gills can be used to diagnose tissue damage caused by benzalkonium chloride. Histopathological examination is a valuable tool for assessing toxicity and indicating exposure to contaminants [10]. Severe damage to the gills can lead to mortality due to the inadequate supply of DO in the water [11].

Therefore, the continuous use of disinfectant (benzalkonium chloride) during the COVID-19 pandemic accumulated in the waters. This impact affects aquatic commodities, including goldfish, an ornamental fish with high sensitivity. Apart from that, Goldfish also supports the economy of the Indonesian people, so their productivity continues to increase. Ornamental fish exports increased from 21 million to 33 million USD from 2012 to 2019 [12, 13]. Benzalkonium chloride has the potential to affect several body proteins, including the cytoskeleton, nervous and endocrine systems, and fish physiology.

This study aimed to determine the effect of benzalkonium chloride as a disinfectant on the level of color brightness, fish growth, gill histopathology, and mortality (fish physiology) in goldfish.

## Materials and Methods

### Ethical approval

The study was approved by the Examiner Committee of the Department of Aquaculture, Faculty of Fisheries and Marine Sciences, Universitas Airlangga (Assignment Letter No. 3727/UN3.1.12/PK/2022). The fish was well cared throughout the study, and their feeding and water quality control were conducted in compliance with the Indonesian National Standard (SNI) 7733:2018.

### Study period and location

The study was conducted for 28 days (August 2022) in the Laboratory of Anatomy and Aquaculture, Faculty of Fisheries and Marine, Universitas Airlangga, Indonesia. While the toxicity test was conducted at the Laboratory of Pharmacology, Faculty of Veterinary Medicine, Universitas Airlangga, Indonesia.

### Research treatment

This study was conducted under five treatment concentrations of benzalkonium chloride on the goldfish (*Carassius auratus*) rearing media: (T1) 0 mg/L, (T2) 0.03 mg/L, (T3) 0.06 mg/L, (T4) 0.09 mg/L, and (T5) 0.12 mg/L. The impact of benzalkonium chloride as a disinfectant was measured by analyzing the color brightness level of goldfish, length-weight relationship, histopathology of the fish gills, and toxicity test.

### Cultivation process

The fish were provided with proper care, and pellet feed was given *ad libitum* at 3–5% of their body weight. Daily monitoring of the water quality was performed, and every second day, half of the water in the tank was replaced while refilling benzalkonium chloride [7]. The sampling of fish gills begins by anesthetizing the fish and then piercing the brain until the fish dies, and then the gills are taken.

### Observation of color brightness levels

Adobe Photoshop CC 2015 software was used to observe the color brightness. The specified fish sample point was taken using the eyedropper tool (I). Then the hue value of the fish can be seen in the set foreground color. Hue values were measured once a week to determine color changes, namely, on days 0, 7, 14, 21, and 28.

### Calculation of fish growth

Absolute length growth can be interpreted as the length of the fish measured from the head to the tail fin [14].

#### The growth of fish length

In analyzing the absolute growth of fish length, it is necessary to calculate it using the formula according to Effendie [15].

#### The growth of fish weight

In analyzing absolute biomass growth, it is necessary to calculate using the formula according to Effendie [16].

#### Length-weight relationship

The length-weight relationship in fish can be analyzed using the Linear Allometric Model equation using the formula according to Fuadi *et al*. [17].

### Preparations and histopathological observations of goldfish gills

Histopathological observations were prepared by dissecting fish to remove gills. The organ was then stored in a sample pot (to put a goldfish gill sample in) with fixation in a 10% formalin solution. Then, a dehydration process was conducted using a solution consisting of 70%, 80%, 90%, 96%, and 100% alcohol, aiming to inhibit the growth of bacteria that will cause decay. Then, xylol was used as an intermediate between the dehydrated solution and infiltration in the clearing process. The sample was infiltrated (blocking) using an embedding set and then given liquid paraffin and cooled. The blocks of organ tissue were cut using a microtome with a thickness of ±6 microns.

Moreover, staining was carried out using preparations that were soaked in xylol solution on the top of the object glass for 5 min each. Then, the preparations were soaked in 100% alcohol for 5 min. Afterward, the preparations were dipped in distilled water and soaked in Harris hematoxylin C_16_H_14_O_6_ for 15 min. The preparations were dipped in distilled water by moving them up and down. Next, the preparations were put into 1% acid alcohol 7–10 times and then soaked in distilled water for 1 min and rinsed. In the following stage, they were dipped in eosin for 2 min. The preparations were dipped in a series of graded alcohols of 96%, 96%, 100%, and 100% of each for 3 min to inhibit the bacterial growth that would cause decay, and absolute ethanol was dehydrated. The preparations were immersed in xylol as an intermediary between the dehydration solution and infiltration twice for 5 min. Then the clearing process was conducted in which the preparations were dried and the mounting media process was carried out. Histological preparations can be observed under a microscope [18].

### Calculation of mortality rates

Mortality observations were conducted weekly, specifically on weeks 1, 2, 3, and 4. The mortality rate was calculated using the formula: (number of fish that died/number of fish that were still alive) × 100%, following the guidelines provided by the EPA [19].

### Toxicity test

The levels of benzalkonium chloride in the fish body were determined using the toxicity test with the GC-MS method. The GC-MS method, which combines gas chromatography and mass spectrometry, is commonly used to analyze and identify different substances in test samples, as recommended by Nakagawa-Izumi *et al*. [20].

### Statistical analysis

The collected data were analyzed statistically by using analysis of variance (ANOVA). Color brightness, growth, and mortality data were further analyzed using Duncan’s test if the results differed significantly. Meanwhile, if the gill histopathology data experienced significantly different results, further tests would be carried out, namely, the Kruskal–Wallis and Mann–Whitney tests.

## Results

### Brightness level of fish color

Data analysis using ANOVA showed that the concentration of benzalkonium chloride on the level of color brightness (Table-1) from day 0 to days 7 and 14 obtained results that were not significantly different (p > 0.05). Meanwhile, on days 21 and 28, the results were significantly different (p < 0.05), so Duncan’s test was needed.

**Table-1:** Hue values of goldfish (*Carassius auratus*) per 7 days for 28 days of cultivation in each treatment.

Treatment	Hue value on day (°)					Color	Detail

	0	7	14	21	28		
T1	10.55 ± 0.91	10.47 ± 0.9	10.12 ± 0.54	9.47 ± 1.3^a^	9.27 ± 1.27^a^	80% Red	Good (positive)
T2	7.89 ± 1.79	9.06 ± 1.98	9.57 ± 1.48	10.65 ± 2.62^ab^	11.55 ± 3.36^ab^	80% Red	Good (positive)
T3	7.6 ± 1.07	9.53 ± 0.71	10.89 ± 1.26	14.78 ± 1.95^b^	16 ± 2^b^	60% Red	Faded (negative)
T4	9.27 ± 2.67	10.87 ± 1.18	12.42 ± 3.42	14.05 ± 4.64^ab^	15.5 ± 5.07^b^	60% Red	Faded (negative)
T5	9.17 ± 2.39	9.5 ± 1.57	12.5 ± 0.5	13.67 ± 0.58^ab^	15.33 ± 1.15^b^	60% Red	Faded (negative)

(T1) 0 mg/L, (T2) 0.03 mg/L, (T3) 0.06 mg/L, (T4) 0.09 mg/L, and (T5) 0.12 mg/L, The growth of goldfish

### Length and weight

Analysis of variance analysis revealed significant differences (p < 0.05) and (p < 0.01) in the impact of benzalkonium chloride concentration on the length and weight of goldfish. To further explore these differences, Duncan’s test was conducted. Figure-2 illustrates the average length and standard deviation (SD) for each treatment.

**Figure-1:**
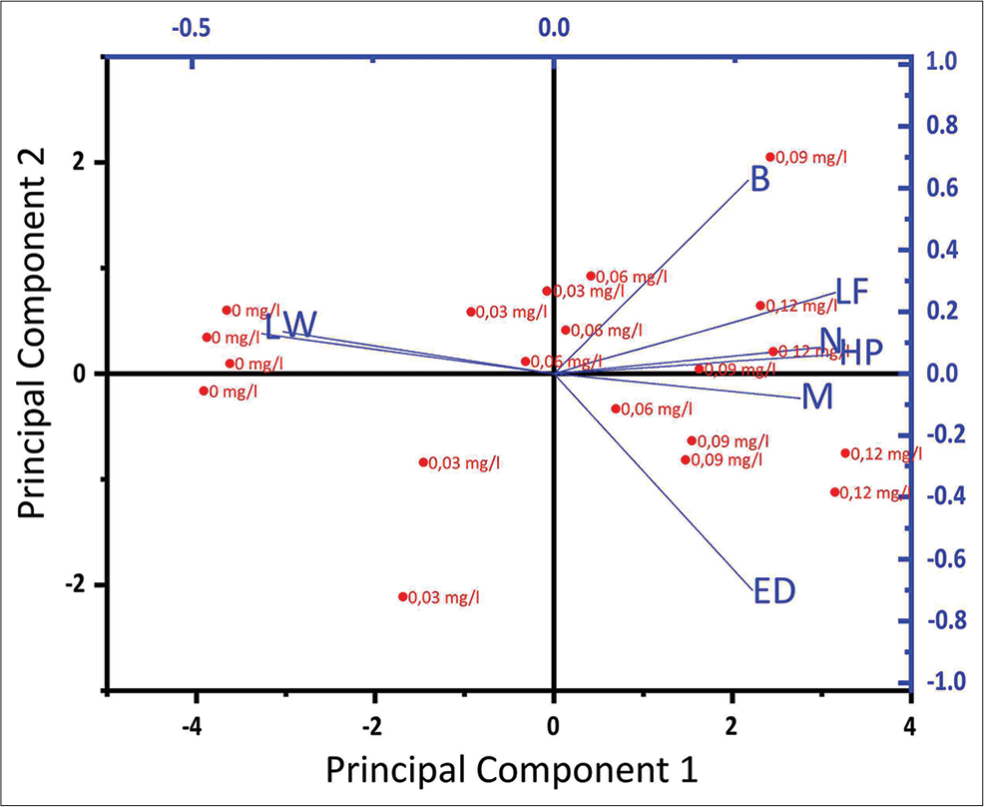
Principal component analysis. W: Weight, L: Length, B: Brightness, M: Mortality, E: Edema, H: Hyperplasia, LF: Lamela Fusion, N: Necrosis.

**Figure-2:**
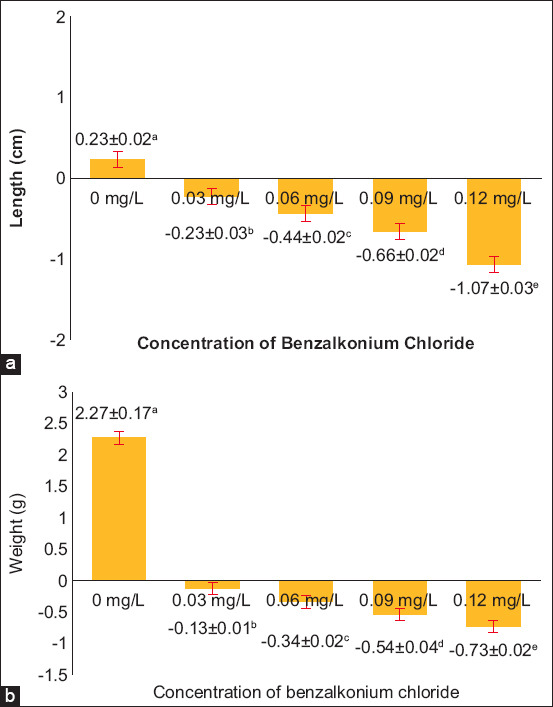
Graph of the (a) length and (b) weight of goldfish in each treatment.

### Length-weight relationships

The relationship between length and weight shows that the value of b (y = ax-b) in the control treatment 0 mg/L (T1) produces positive allometric growth, whereas at a concentration of 0.03 mg/L (T2), 0.06 mg/L (T3), 0.09 mg/L (T4), and 0.12 mg/L (T5) resulted in negative allometric growth.

### Histopathology of goldfish gills

The highest average percentage of gill tissue damage was at T4 (0.12 mg/L) and the lowest was at T1 (0 mg/L). Additional tests using Mann–Whitney showed that the lowest value of damage was at T1 (0.0 mg/L) which was significantly different from 0.03 mg/L (T2), 0.06 mg/L (T3), 0.09 mg/L (T4), and 0.12 mg/L (T5). The results showed that the higher the concentration of benzalkonium chloride, the higher the level of damage to the fish gills.

### Mortality

The results of goldfish mortality were significantly different. The highest percentage of fish mortality occurred at a concentration of 0.12 mg/L. This is due to toxic compounds from benzalkonium chloride which can interfere with the fish’s respiratory system. Mortality at a concentration of 0.12 mg/L results in 85% mortality with a standard deviation of 0.5. The lowest percentage of fish mortality occurred at concentrations of 0.03 mg/L and 0.06 mg/L.

### Toxicity test

The results of the toxicity test on goldfish without benzalkonium chloride T1 (0 mg/L) were 90 Quality. Exposure to benzalkonium chloride was 91 Quality. Benzalkonium chloride contains the dodecane compound, 1-chloro- which is a part of the mixed compounds of benzalkonium chloride. The process of benzalkonium chloride can enter through the surface of the gill skin. Gills are organs that are susceptible to the effects of chemicals and become target organs for the effects of toxic chemicals or toxins. So that the impact of gill tissue damage caused by benzalkonium chloride will result in death of organisms and indirectly have a negative impact on the environment because it causes water pollution.

### Water quality

The average water quality of the goldfish research was as per the following; temperature 25ºC–28.6ºC, dissolved oxygen (DO) 5.14–7.7 ppm, acidity degree pH 6.9–7.9, and ammonia 0. Fish maintenance in all treatments still have tolerance levels.

### Principal component analysis (PCA) test

Principal component analysis is defined as a multivariate (multivariable) data selection technique that has the utility of finding a small set of linear combinations of uncorrelated covariates and ensuring that the selected linear combinations capture a large amount of variance (Figure-1).

## Discussion

Based on Table-1, the brightness level of fish color during the 28 days of cultivation in the T1 control treatment produced a hue value of 9.27° (80% red color level), which decreased gradually each day, indicating an improvement (positive) in the fish color. This finding was consistent with Sari’s [21] study, which stated that the lower the hue value, the better (positive) the fish color. On the other hand, in T2, T3, T4, and T5, the hue values were 11.55° (80% red color level), 16° (60% red color level), 15.5° (60% red color level), and 15.33° (60% red color level), respectively, which increased gradually each day, indicating that the color on the fish body was fading (negative). In this study, it can be seen that the hue values with the highest color change were found in T3 (0.06 mg/L), while the hue values with the lowest color change were found in T2 (0.03 mg/L).

Signal stimulation originating from benzalkonium chloride in the environment brings specific activator-inhibitor signals that interfere with fish body proteins to nerve cells. Pigment cells can form pigment patterns in the nervous system (neural). The pigment pattern undergoes color changes in the brain’s dendrogram and skin protein profile. In this process, there is a change in the distribution (migration) and number of pigment cells in the integumentary system. One of the factors that affect the color of the goldfish’s body is genetic factors. The presence of benzalkonium chloride during cultivation can interfere with body proteins, affecting the genetics of fish. This is reinforced by Yahyadi’s statement [22], suggesting that different environmental conditions can completely affect fish genetics and the fish’s body color.

Based on Figure-2, the length and weight of the fish during the 28-day cultivation period showed an increase in length, where the control treatment of 0 mg/L (P1) resulted in a length of 0.23 and a weight of 2.27. Meanwhile, concentrations of 0.03 mg/L (P2), 0.06 mg/L (P3), 0.09 mg/L (P4), and 0.12 mg/L (P5) resulted in a length of −0.23; −0.44; −0.66; −1.07, respectively, and a weight of 2.27; −0.13; −0.34; −0.54; −0.73, respectively, indicating that fish have decreased or shrunk due to benzalkonium chloride contamination during fish cultivation. Exposure to benzalkonium chloride disrupts the cytoskeleton in cell activity, increasing the number of stress fibers containing actin. These triggerfish to experience chemical and physiological stress in the environment, which can stop the growth of fish body cells [23].

**Figure-3:**
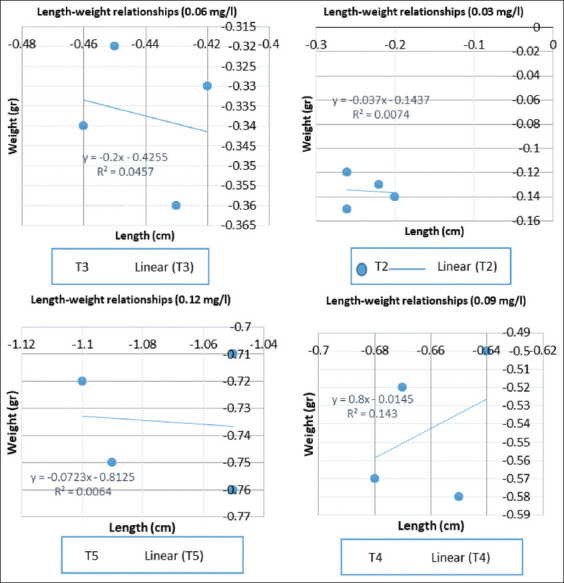
Graph of length-weight relationships of goldfish.

Based on the graph of the relationship between length and weight above (Figure-3), the relationship between length and weight is indicated by the value of b (regression coefficient) from the regression analysis. In this study, it was found that the highest value of b was found at T4 (0.09 mg/L) of 0.8, while the lowest value of b was found at P2 (0.03 mg/L) of −0.037. Good growth can be seen from a high b value. The b value at T1 shows b > 3, which means that the fish in this treatment was fat. Meanwhile, at T2, T3, T4, and T5, the fish was thin. This is reinforced by Effendie’s statement [24] that the b value for treatment, if b = 3, indicates that the growth in length of the fish is balanced with the weight of the fish, called isometric growth. Meanwhile, if the value of b < 3 or b > 3 is called allometric growth. B < 3’s value is negative allometry, indicating that the fish is thin, where the growth in length is faster than the weight of the fish. Meanwhile, the value of b > 3 is positive allometry, indicating that the fish is fat, where the growth of the fish’s weight is faster than the length of the fish.

**Figure-4:**
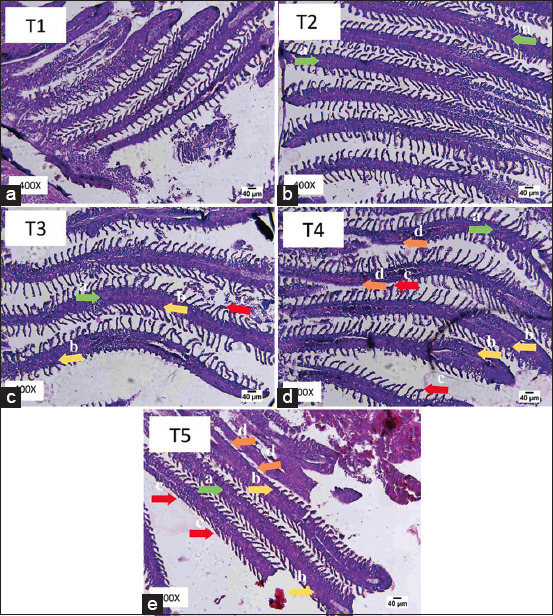
Histopathological description of primary and secondary lamella of goldfish exposed to benzalkonium chloride. Note: (a) Edema 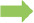, (b) Hyperplasia 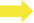, (c) Lamella Fusion 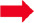, (d) Necrosis
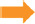

Based on observations on the structure of the goldfish gill tissue in Figure-4 and Table-2, there were significantly different results between the control treatment (T1) and the treatments exposed to benzalkonium chloride (T2, T3, T4, and T5). The condition of goldfish gills exposed to benzalkonium chloride with concentrations of (T2) 0.03 mg/L, (P3) 0.06 mg/L, (T4) 0.09 mg/L, and (T5) 0.12 mg/L showed an appearance of abnormal gill tissue with some damage to the primary and secondary lamellae of the goldfish gills for 4 weeks. The highest scoring value (T5) indicated the most severe damage at 0.12 mg/L of 10. Cells that experience necrosis will detach from their supporting tissues after the blood supply is lost, which encourages proliferation for new cell turnover [25]. The characteristics of necrotic tissue are that the color is paler than the normal color, there is a loss of color range, and the cells fade as the nucleus shape shrinks and the cytoplasm is lost, making it unable to absorb the dye given in the process of making histopathological preparations [26]. Changes in the microanatomical structure of the gills can be used as an indicator of environmental pollution, starting from the occurrence of contamination, mild pollution to severe pollution [27].

**Table-2:** Average percentage of edema + SD, hyperplasia + SD, lamella fusion + SD, and necrosis+SD in each histopathological treatment of goldfish gills.

Treatment	Edema ± SD	Hyperplasia ± SD	Lamella Fusion ± SD	Necrosis ± SD
T1	0 ± 0^a^	0 ± 0^a^	0 ± 0^a^	0 ± 0^a^
T2	1 ± 1.4^b^	0.5 ± 0.7^b^	0.5 ± 0.7^b^	0.5 ± 0.7^b^
T3	1 ± 0^b^	2 ± 0^b^	1 ± 0^b^	0.5 ± 0.7^b^
T4	1.5 ± 0.7^b^	2 ± 0^b^	1.5 ± 0.7^b^	2 ± 0^b^
T5	2 ± 1.4^b^	2.5 ± 0.7^b^	2 ± 0^b^	2 ± 0^b^

SD=Standard deviation, Notation (a, b) showing significancy of the treatment effect (p < 0.05).

Based on the data, the results of goldfish mortality in Table-3 showed a significant difference between the control treatment (T1) and the treatment exposed to benzalkonium chloride (T2, T3, T4, and T5). The highest percentage of fish mortality occurred at a concentration of 0.12 mg/L. This is due to the presence of toxic compounds from benzalkonium chloride, which can interfere with the fish respiratory system. Mortality at a concentration of 0.12 mg/L resulted in 85% mortality with an SD of 0.5a. The lowest percentage of fish mortality occurred at concentrations of 0.03 and 0.06 mg/L. Fish’s mortality occurred due to exposure to benzalkonium chloride. According to Kamiswari *et al*. [28], the higher the concentration, the greater the pH value in water because the surfactant carbon chain can bind oxygen from air to water. Therefore, the oxygen in the cultivation ponds is toxic to fish. A Toxicant is a substance that causes injury, illness, or death to an organism, usually through chemical reactions or other activities on a molecular scale. Toxic ability, among others, is influenced by the physical properties of the chemical, dose, toxic concentration that enters the body, length of exposure, type of compound, toxic pathways in the body, and host factors [29].

**Table-3:** Treatment concentration and average percentage of mortality + SD.

Treatment	Average percentage of mortality + SD
T0	25 ± 0.5^a^
T1	60 ± 1.41^b^
T2	60 ± 1.41^b^
T3	80 ± 0^b^
T4	85 ± 0.5^b^

SD=Standard deviation

Based on Table-4, toxicity test samples from the treatments exposed to benzalkonium chloride were taken randomly to produce the compound dodecane, 1-chloro-. Dodecane, 1-chloro-, is a compound of the benzalkonium chloride composition. This is supported by Koyama and Shimazu [30], who stated that benzalkonium chloride is a mixture of quaternary ammonium salts showing the structure [C_6_H_5_CH_2_ (CH_3_)2R] Cl, where R is a mixture of C_8_H_17-_C_18_H_37_. However, the main structures of R are benzyldimethyldodecylammonium C_12_H_25_ (C_12_) bromide, benzyldimethyl-tetradecylammonium C_14_H_29_ (C_14_) chloride, and benzyldimethylhexadecylammonium C_16_H_33_ (C_16_) chloride, C_12_-40%, C_14_-20%, and C_12_ plus C_14_-70%.

**Table-4:** Toxicity test by random sample to control treatment and exposure to benzalkonium chloride.

No.	Treatment	Compound	Quality
1	Control	Caryophyllene	90
2	Exposure to benzalkonium chloride	Dodecane, 1-chloro-	91

Water quality during cultivation is a physical and chemical factor that can be directly measured and can influence the living environment of fish. Parameters that measured the quality of goldfish water in this study included pH, temperature, DO, and ammonia (NH_3_). Based on Table-5, the results of the water quality study show the results of tolerance during fish cultivation in treatments T1, T2, T3, T4, and T5. The normal limits for goldfish water quality in a good environment include a temperature range of 23°C–29°C, DO of 5.0–8.0 ppm, pH of 6.5–8.0, and ammonia of 0.00–0.15 ppm [31]. The values of environment exposed to disinfectants based on the Fish Cultivating Business Unit that is Certified by Marine and Fisheries Ministry, Republic of Indonesia for the Implementation: 27°C–28°C, pH of 6.3–7.0, 5.0–6.0 ppm, and ammonia of 0.0–0.2 ppm.

**Table-5:** Water quality during the cultivation of goldfish (*Carassius auratus*).

Treatment	Parameter observed			

	Temperature (°C)	pH	DO (mg/L)	Ammonia (mg/L)
T1	26.1–28	7.3–7.8	5.14–6.82	0
T2	26.3–28	7.3–7.9	5.81–7.7	0
T3	25.8–28	7.3–7.9	5.59–6.23	0
T4	25.6–28.1	7.1–7.9	5.37–6.55	0
T5	25.6–28,6	6.9–7,9	5.26–5.37	0

The PCA results show that mortality has a correlation with the appearance of edema; fish brightness correlates with lamella fusion, necrosis, and hyperplasia, while weight and length are correlated with each other. These results are in accordance with research [32] that toxic affects the physiological, histomorphological, and growth of fish. Benzalkonium chloride is a biocidal active substance and can kill pathogenic microorganisms. LC_50_ and EC_50_ show that chlorine toxicity affects non-target organisms. The no observed effect concentration of a chlorine-based biocide depends on the life stage and species of animal tested, the type of biocide, time of exposure, and the nature of the water (seawater or freshwater). This effect can affect death, growth, and reproduction in aquatic biota.

## Conclusion

Based on the research conducted on the impact of benzalkonium chloride on goldfish, it can be concluded that the concentration of benzalkonium chloride significantly affects the brightness level of fish color. On days 21 and 28 of observation, the highest hue value of 16° was recorded at T3 (0.06 mg/L), indicating a more pronounced color change. Conversely, the lowest hue value was observed at T2 (0.03 mg/L) with a value of 11.55°. Furthermore, the concentration of benzalkonium chloride also affected the growth of goldfish. The highest b value of 0.8 was found at T4 (0.09 mg/L), indicating favorable growth conditions. On the other hand, the lowest b value of −0.04 was recorded at T2 (0.03 mg/L), indicating a less optimal growth pattern. In addition, the histopathological analysis revealed that concentrations of 0.03 mg/L (T1), 0.06 mg/L (T2), 0.09 mg/L (T3), and 0.12 mg/L (T4) of benzalkonium chloride caused damage to the gill tissue of goldfish. The histopathological analysis revealed several types of damage in the gill tissue of goldfish exposed to benzalkonium chloride, including edema, hyperplasia, lamella fusion, and necrosis. These damages indicate the detrimental effects of benzalkonium chloride on the fish’s gill health of the fish. Furthermore, the study also found a correlation between benzalkonium chloride exposure and the mortality rates of goldfish. The lowest percentage of mortality, recorded at concentrations of 0.03 mg/L and 0.06 mg/L, was 60%. In contrast, the highest percentage of mortality, observed at a concentration of 0.12 mg/L, reached 85%. Despite the exposure to benzalkonium chloride, the water quality during goldfish cultivation remained within normal limits.

## Authors’ Contributions

SRA and FAS: Conducted the research and drafted the manuscript. ALN, SA, and NM: Commented and advised on the experiment and revised the manuscript. LAS: Designed the study, analyzed the data, and revised and edited the manuscript. All authors have read, reviewed, and approved the final manuscript.

## References

[1] World Health Organization (2020). Water, Sanitation, Hygiene and Waste Management for the COVID-19 Virus.

[2] Prasetiya F.S, Destiarani W, Nuwarda R.F, Rohmatulloh xF.G, Natalia W, Novianti M.T, Ramdani T, Agung M.U.K, Arsad S, Sari L.A, Pitriani P, Suryanti S, Gumilar G, Mouget J.L, Yusuf M (2023). The nanomolar affinity of C-phycocyanin from virtual screening of microalgal bioactive as potential ACE2 inhibitor for COVID-19 therapy. J King Saud Univ Sci.

[3] Subpiramaniyam S (2021). Outdoor disinfectant sprays for the prevention of COVID-19:Are they safe for the environment?*Sci*. Total Environ.

[4] Faziara A, Ekhtelat M (2012). The disinfectant effects of benzalkonium chloride on some important foodborne pathogens. Am Eurasian J Agric Environ Sci.

[5] NIER (National Institute of Environmental Research in Korea) (2016). Risk Assessment of Pharmaceuticals with Potential Ecological Risks. NIER-SP2016-2221.

[6] Hora P, Pati S.G, Mcnamara P.J, Arnold W.A (2020). Increased use of quaternary ammonium compounds during the SARS-CoV-2 pandemic and beyond:Consideration of environmental implications department of civil, environmental, and geo-engineering, university of Minnesota-twin. Environ. Sci. Technol. Lett.

[7] Kwon Y.S, Jung J, Kim Y.J, Park B, Shon J.C, Kim J.H, Park J, Kim S.G, Seo J.U (2020). Proteomic analysis of whole-body responses in medaka (*Oryzias latipes*) exposed to benzalkonium chloride. J. Environ. Sci. Health A Tox Hazard. Subst. Environ. Eng.

[8] Kusumawati D, Permana S, Setiawati K.M, Haryanti (2012). The role of the Aim1 gene and light intensity on the pigment pattern characters of the black clownfish (*Amphiprion percula*). J Ris. Aquacult.

[9] Yu D, Ji C, Zhao J, Wu H (2016). Proteomic and metabolomic analysis on the toxicological effects of as (III) and as (V), Juv. Mussel *Mytilus galloprovincialis*. Chemosphere.

[10] Cengiz E.I (2006). Gill and kidney histopathology in the freshwater fish *Cyprinus carpio* after acute exposure to deltamethrin. Environ. Toxicol. Pharmacol.

[11] Billah R.A (2020). Effect of Majapahit Fruit Extract (*Crescentia cujete*) on Mortality and Leukocyte Differential of Catfish (*Clarias batrachus*) Post challenge Test with *Aeromonas hydrophyla* Bacteria.

[12] Kementerian Kelautan dan Perikanan [KKP] (2021). ORNAMENTAL Fish Cultivation Increases People's Income Amidst a Pandemic. Directorate General of Aquaculture.

[13] Sari R.E.R, Tjahjaningsih W, Kismiyati K (2019). Histopathological changes in comet fish (*Carassius auratus auratu*s) skin tissue due to *Argulus japonicu*s infestation [Perubahan histopatologi jaringan kulit ikan komet (*Carassius auratu*s auratus) akibat infestasi *Argulus japonicus*]. JAFH.

[14] Dwiardani K.H, Sari L.A, Sari P.D.W, Nindarwi D.D, Arsad S (2020). The effect of feed larvae *Chironomus* spp. and high pellet protein to seedling goldfish (*Carassius auratus*). IOP Conf. Ser. Earth Environ. Sci.

[15] Effendie M.I (2004). Introduction Aquacult. Self-Help Spreader.

[16] Effendie M.I (1997). Fish Biology Method. Dwi Sri Foundation.

[17] Fuadi Z, Dewiyanti I, Purnawan S (2016). Long relationship of weight of fish caught in Krueng Simpoe, Bireuen District, Aceh. J. Ilmiah Mahasiswa Kelautan Perikanan Unsyiah.

[18] Kusumadewi M.R, Suyasa I.W.B, Berata I.K (2015). Bioconcentration Levels of Heavy Metals and Hispathological Features of Mujair fish (*Oreochromis mossambicus*) Living in Tukad.

[19] Environmental Protection Agency (EPA) (1996). Ecological Effects Test Guidelines. Freshwater and Marine:Fish Acute Toxicity Test.

[20] Nakagawa-Izumi A, H'ng Y.Y, Mulyantara L.T, Maryana R, Do V.T, Ohi H (2017). Characterization of syringyl and guaiacyl lignins in thermomechanical pulp from oil palm empty fruit bunch by pyrolysis-gas chromatography-mass spectrometry using ion intensity calibration. Ind. Crops Prod.

[21] Sari O.V, Hendrarto B, Soedarsono P (2014). The effect of variations in food types on nemo coral fish (*Amphiprion ocellaris Cuvier*, 1830) in terms of changes in color, growth, and survival rates, Diponegoro. J. Maquares Manag. Aquat. Resour.

[22] Yahyadi Y, Aliah R.S, Murdjan M, Sumantadinata K (2004). The correlation between the number of spot body length in humback grouper, *Cromileptes altivelis*. J. Aquac. Indones.

[23] Nirmala K, Yani H, Riza P.W (2012). The addition of salt in the water media containing zeolite and active charcoal on closed system transportation of gourami fish fry *Osphronemus goramy* Lac. J. Aquac. Indones.

[24] Effendie M.I (2002). Fisheries Biology.

[25] Winaruddin E, Aliza D, Budiman H (2013). Effect of population density on anatomical and histopathological pathology of tilapia (*Oreochromis niloticus*) gills. Veterinary haqqawiy, Janni. Med. J.

[26] Edwin T, Ihsan T, Amas R.L (2018). Tissue Changes in Tilapia Gills Due to Exposure to Leather Tannery Liquid Waste Proceedings of the National Seminaron Environmental Science and Technology III.

[27] Abdel-Latif H.M.R, Khashaba A.M.A (2017). Subchronic toxicity of Nile tilapia with different exposure routes to *Microcystis aeruginosa*:Histopathology, liver functions, and oxidative stress biomarkers. Vet. World.

[28] Kamiswari R, Hidayat M.T, Rahayu Y.S (2013). The Effect of Detergent Giving on the Mortality of *Platy* spp.

[29] Jin M.H, Hong C.H, Lee H.Y, Kang H.J, Han S.W (2010). Toxic effects of lactational exposure to 2,3,7,8-tetrachlorodibenzo-p-dioxin (TCDD) on development of male reproductive system:Involvement of antioxidants, oxidants, and p53 protein. Environ. Toxicol.

[30] Koyama K, Shimazu Y (2005). Benzalkonium Chlorides.

[31] Johan C.A.C, Zainathan S.C (2020). Megalocytiviruses in ornamental fish:A review. Vet. World.

[32] Tavares-Dias M (2021). Toxic, physiological, histomorphological, growth performance and antiparasitic effects of copper sulphate in fish aquaculture. Aquaculture.

